# Simultaneous Bilateral Patellar Tendon Rupture in a Young Adult Male: A Case Report and Review of the Literature

**DOI:** 10.7759/cureus.10649

**Published:** 2020-09-25

**Authors:** Adrian Moy, Ethan Song, Sean J Wallace, Robert Teixeira, Daniel Torres

**Affiliations:** 1 Orthopedic Surgery, University of South Florida Morsani College of Medicine, Tampa, USA; 2 Plastic Surgery, University of South Florida Morsani College of Medicine, Tampa, USA; 3 Department of Surgery - Division of Plastic & Reconstructive Surgery, Lehigh Valley Health Network, Allentown, USA; 4 Department of Surgery - Division of Orthopedic Surgery, Lehigh Valley Health Network, Allentown, USA

**Keywords:** patellar tendon rupture, bilateral, tendonitis, osgood-schlatter, knee extensor mechanism

## Abstract

A bilateral patellar tendon rupture is extremely rare and has only been documented in case reports. Although the etiology remains unknown, predisposing factors include steroid usage, systemic diseases, and tendinopathies. In the present case, a healthy 33-year-old male with a prior history of bilateral patellar tendonitis and a diagnosis of Osgood-Schlatter disease during adolescence experienced simultaneous bilateral patellar tendon rupture after playing volleyball. He underwent bilateral patellar repair without complications. In the absence of trauma, spontaneous bilateral patellar tendon ruptures are associated with several predisposing factors, including systemic diseases, prior corticosteroid or fluoroquinolone usage, and history of tendinopathy. Injuries can be classified based on the location of the rupture. Bilateral patellar tendon ruptures can be misdiagnosed due to the rarity of cases and the lack of a normal comparative knee. Radiographic techniques can aid in the diagnosis, leading to early surgical treatment and improved outcomes. Early diagnosis and prompt surgical repair contribute to good functional outcomes in this potentially debilitating injury pattern.

## Introduction

Bilateral ruptures of the patellar tendons are extremely rare and have only been documented in case reports [1-3]. Most cases have been associated with corticosteroid and/or fluoroquinolone use and systemic diseases, including systemic lupus erythematosus and rheumatoid arthritis [4]. Additionally, long-term microtrauma and existing tendinopathy may also contribute to bilateral rupture. According to Davidson's theory, multiple recurrent microtears within the tendon substance have been attributed as a cause of rupture [2].

A bilateral rupture in the absence of any systemic disease or corticosteroid use is exceedingly rare and only accounts for a small percentage of the reports in the literature. We describe a case of bilateral patellar tendon rupture sustained following minimal trauma by an otherwise healthy patient with a history of bilateral patellar tendonitis and Osgood-Schlatter (OS) disease.

## Case presentation

An otherwise healthy 33-year-old male with a history of bilateral patellar tendonitis and OS presented to a level-one trauma center after sustaining bilateral knee trauma. He was playing volleyball and felt a snap and heard a popping sound while jumping after having both knees in flexion. He sustained a ground-level fall and subsequently could not ambulate and immediately noticed a loss of bilateral knee extension. He denied any significant orthopedic or rheumatologic family medical history and had no recent antibiotic, steroid, or medication usage.

Physical exam revealed a fit male with easily palpable defects along the superior poles of both the left and right patellae. There was diffuse surrounding soft tissue edema over both knee joints. The patient was unable to perform a straight leg raise on either side or extend either leg. There were no signs of neurovascular compromise. All laboratory values were unremarkable, including a normal vitamin D level.

**Figure 1:**
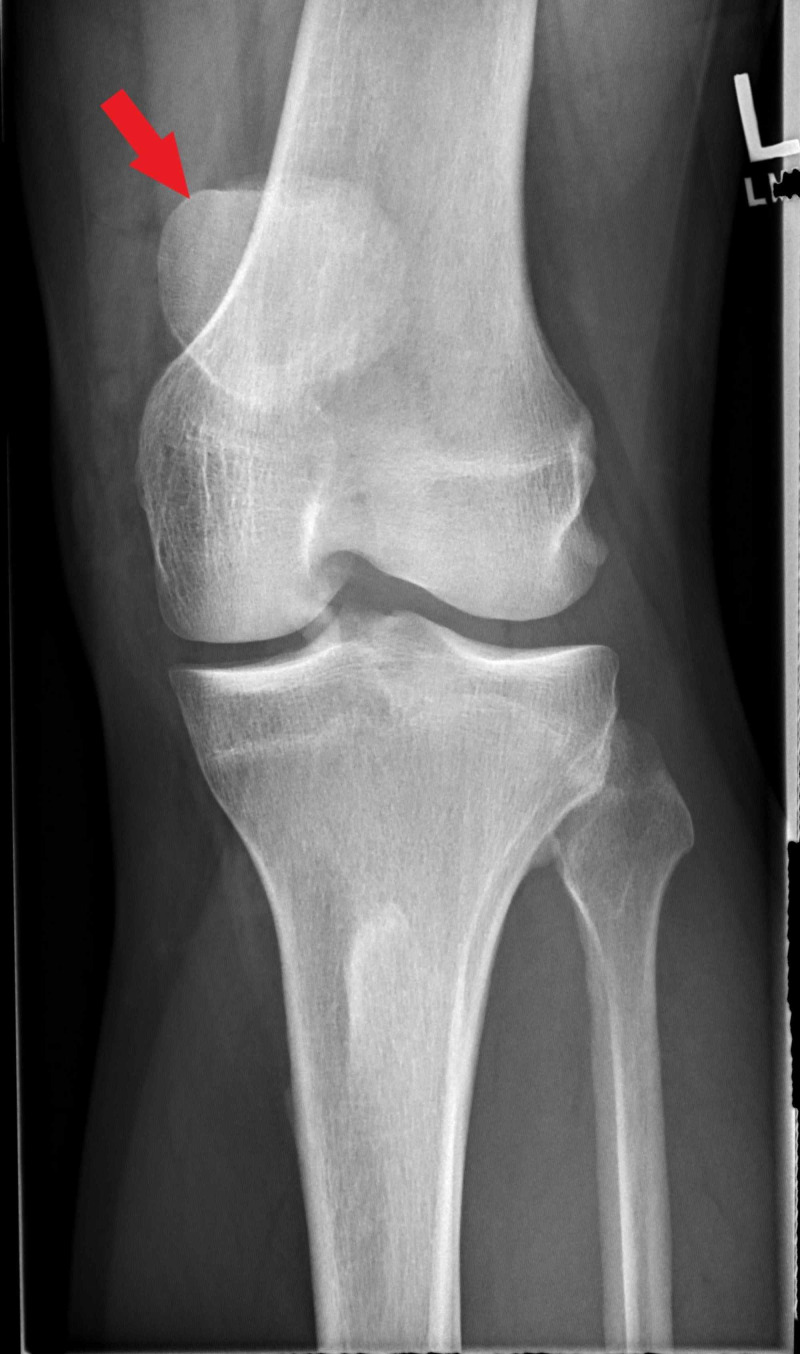
Anterior-posterior view radiograph of the left knee demonstrating high-riding patella (red arrow)

**Figure 2:**
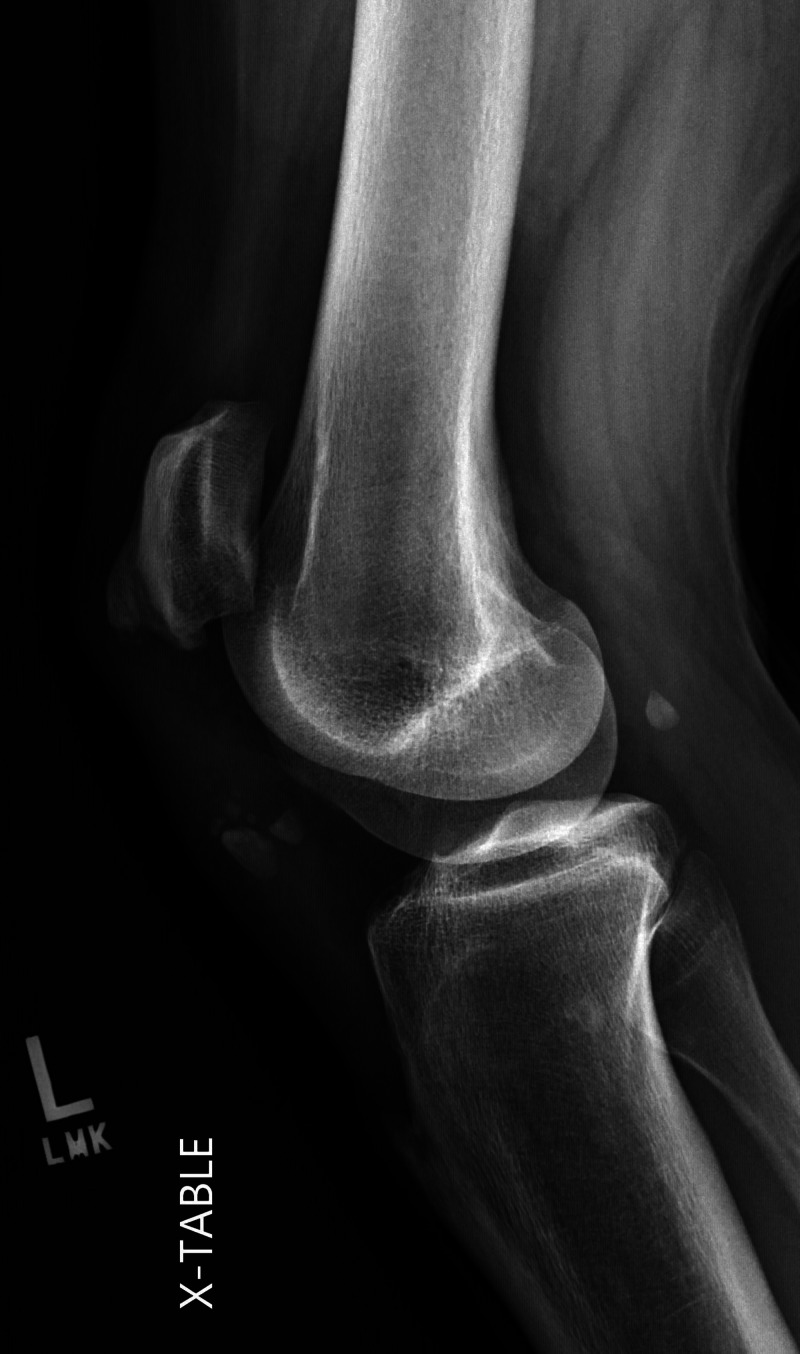
Lateral view radiograph of the left knee

**Figure 3:**
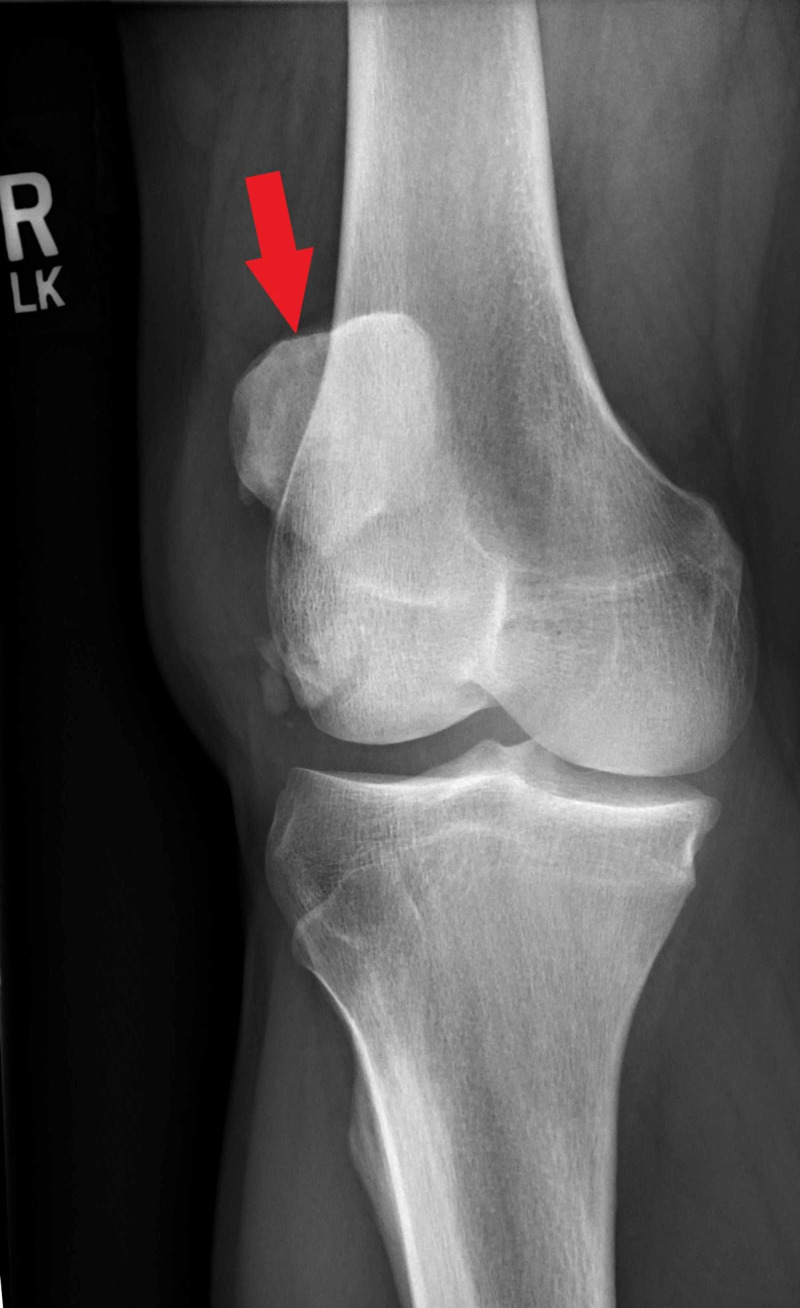
Anterior-posterior view radiograph of the right knee demonstrating high-riding patella (red arrow)

**Figure 4:**
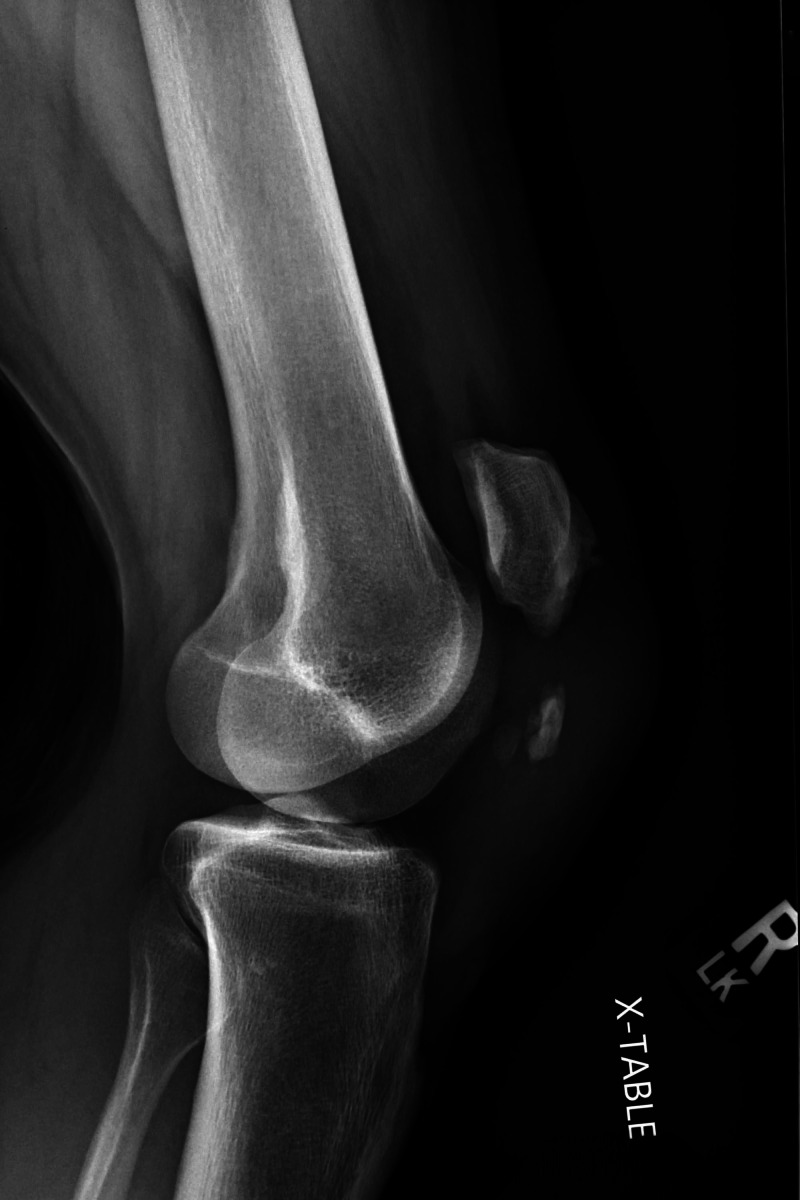
Lateral view radiograph of the right knee

Plain radiographs revealed high-riding patellas bilaterally and increased distance between the tibial tubercle and patella. There was notable central ossification of both patellar tendons, indicative of chronic injury (Figures 1-4). Based on clinical evidence and confirmed radiologically, the patient was diagnosed with closed acute bilateral patellar tendon ruptures.

The patient underwent semi-urgent bilateral patellar tendon repair without complications via midline incisions over bilateral knee joints. Torn patellar tendon remnants were identified and a #5 FiberWire (Arthrex, Munich, Germany) suture was used to create two Krackow sutures into the torn distal segment. Four total drill holes were created into the patellar from the inferior to the superior pole. A suture was then passed through to reduce the patellar tendon onto the inferior pole of the patella. The torn medial and lateral retinaculum was repaired with #5 FiberWire sutures. At the completion of the procedure, excellent stability of the patella, along with an adequate range of motion of the knee joints, were achieved.

The patient was discharged on postoperative day three after working with inpatient physical therapy. He has since continued a home physical therapy regimen with a progressive return to baseline function and activities of daily living.

## Discussion

Patellar tendon injuries are generally seen in patients younger than 40 years old and are more common in men [5]. Extensor mechanism injuries are often due to trauma, though bilateral extensor tendon injuries are more commonly reported to be spontaneous, with spontaneous defined as ruptures occurring during movements and activities that should not and usually do not cause musculotendinous damage [1]. Nevertheless, bilateral disruptions of the knee extensor mechanism are a rare entity and bilateral rupture of the patellar tendons are an even rarer subset of cases [6].

The current literature categorizes bilateral patellar tendon ruptures into three etiologies: systemic disease, use of corticosteroids (both oral and injectable), and chronic microtrauma [7]. The category of systemic disease includes conditions such as systemic lupus erythematosus, rheumatoid disease, diabetes mellitus, and hyperparathyroidism. These systemic diseases cause chronic inflammation and amyloid deposition that affect tendon structure and predispose the tendons to rupture [7-8]. Within the second category, corticosteroid use is believed to degrade the tendon through a decreased blood supply and altered collagen synthesis [9]. The third category refers to histologic findings of inflammatory and degenerative changes in spontaneously ruptured tendons suspected to be attributed to repetitive microtrauma. These histologic changes result in the weakening of the tendon, thus predisposing rupture [2,10-11]. In addition to these three main categories, several case reports have described of fluoroquinolone-associated bilateral patellar tendon rupture [3-4,12].

In the present case, the patient did not have a history of systemic disease, corticosteroid use, or fluoroquinolone use. However, the patient did have bilateral patellar tendonitis during his teenage years. The presence of prior tendonitis and continual physical activity as a competitive volleyball player suggests that the patient could have had chronic microtrauma to his patellar tendons, placing him in the third category of etiologies; however, no histological studies were performed to confirm the presence of chronic microtrauma. Interestingly, there was a remote history of bilateral OS with the left knee significantly worse than the right. OS has been reported to be causative of patellar tendon avulsion at the tibial tubercle insertion, though this has not been reported in the literature to contribute to bilateral patellar tendon rupture [13]. Additionally, the patient’s mechanism of injury occurring during a jumping movement is consistent with spontaneous patellar tendon ruptures [1].

Patellar tendon ruptures can be classified by their location of rupture. A type 1 rupture occurs at the origin of the tendon at the inferior pole of the patella. Type 2 ruptures occur as a midsubstance tear through the tendon. Type 3 ruptures are located at the insertion of the patellar tendon to the tibial tubercle [7]. The patient in this case report suffered avulsions of the tendons at the inferior patellar poles on both knees, rending a classification of bilateral type 1 injuries.

Bilateral patellar tendon rupture is a surprisingly commonly missed diagnosis [14]. On evaluation, patients with a patellar tendon rupture present with weakness of knee joint extensions, inability to walk, and pain. An infrapatellar soft-tissue defect may be palpable, along with a high-riding patella bone, known as the patella alta. However, it is possible to retain some flexor ability if the medial and lateral retinacula remain intact. Additional, significant effusion of the knee joint may obstruct the palpable infrapatellar divot and obscure the patella alta [1]. Plain radiographs and ultrasound can help confirm the diagnosis [15]. Previous authors have recommended determining each patella’s position by a lateral radiographic assessment of the Insall-Salvati ratio [16]. This technique can be used to avoid missing bilateral patella tendon ruptures due to a lack of comparison to a healthy contralateral knee.

Treatment of bilateral patellar tendon ruptures is similar to that of single patellar tendon ruptures. Primary tendon repair should be performed promptly, as in this case. Early tendon repair can prevent proximal patella tendon retraction, scarring, and diminished long-term function [16]. Primary tendon repair is commonly performed via suture repair on a healthy tendon with some authors advocating the use of wire cerclage on degraded tendons [14,17]. Postoperative rehabilitation and therapy is generally recommended, but there is no current consensus or standard therapy regimes [7].

## Conclusions

We report a case of spontaneous bilateral patellar tendon rupture in an adult male with no predisposing systemic disease, steroid use, or fluoroquinolone use. The patient has a history of both bilateral patellar tendonitis and Osgood-Schlatter disease, both of which may have contributed to microtrauma and tendon stress and predisposed the patellar tendons to rupture. This patient was discharged three days after surgery with good functional outcomes. Thorough evaluation, early diagnosis, and prompt primary tendon repair are the key components to providing the best chance at improved functional outcomes in patients with spontaneous bilateral patellar tendon ruptures.
